# Meta-Transcriptomic Analysis Reveals Novel RNA Viruses in Polychaetes *Perinereis*

**DOI:** 10.3390/vetsci11060273

**Published:** 2024-06-15

**Authors:** Jingfei Luo, Fan Zhang, Chengyan Zhou, Fanzeng Meng, Guohao Wang, Liang Qiu, Weifeng Shi, Jie Huang, Xuan Dong

**Affiliations:** 1 College of Fisheries and Life Science, Shanghai Ocean University, Shanghai 201306, China; hanye19937593900@163.com (J.L.); noyamelvin54@outlook.com (C.Z.); wgh15866019360@163.com (G.W.); huangjie@ysfri.ac.cn (J.H.); 2 State Key Laboratory of Mariculture Biobreeding and Sustainable Goods, Yellow Sea Fisheries Research Institute, Chinese Academy of Fishery Sciences, Laboratory for Marine Fisheries Science and Food Production Processes, Qingdao Marine Science and Technology Center, Key Laboratory of Maricultural Organism Disease Control, Ministry of Agriculture and Rural Affairs, Qingdao Key Laboratory of Mariculture Epidemiology and Biosecurity, Qingdao 266071, China; zhfan1205@gmail.com (F.Z.); m190100132@163.com (F.M.); qiuliang@ysfri.ac.cn (L.Q.); 3 Shanghai Institute of Virology, Shanghai Jiao Tong University School of Medicine, Shanghai 200025, China; shiwf@ioz.ac.cn; 4 Jiangsu Shufeng Aquatic Seed Industry Co., Ltd., Gaoyou 255654, China

**Keywords:** RNA viruses, *Perinereis*, meta-transcriptomic, virome, aquaculture

## Abstract

**Simple Summary:**

Viruses present a significant challenge to the sustainable growth of the aquaculture sector. *Perinereis*, a valuable live-feed source in aquaculture, carries the risk of harboring unknown viruses, potentially jeopardizing the biosecurity of aquatic organisms. Despite this risk, there is a notable absence of research investigating novel viruses in *Perinereis* using meta-transcriptomic sequencing techniques. Therefore, it becomes imperative to explore the viral diversity within *Perinereis*. In this report, 12 previously unidentified viruses was documented in two distinct *Perinereis* species through meta-transcriptomic sequencing. These newly identified viruses were classified into four major viral families, shedding light on the intricate viral landscape within *Perinereis*. By unveiling these novel viruses, this research contributes to a broader knowledge base of the viral ecology in *Perinereis*, offering valuable insights into the potential risks and impacts associated with *Perinereis* in aquaculture settings. Such findings also highlight the need for enhancing biosecurity measures and disease management strategies within the aquaculture industry.

**Abstract:**

*Perinereis* species are essential benthonic animals in coastal ecosystems and have significant roles as live feed in aquaculture, owing to their high-protein and low-fat nutritional profile. Despite their ecological importance, the viral communities associated with these organisms need to be better understood. In this study, we generated 2.6 × 10^8^ reads using meta-transcriptomic sequencing and *de novo* assembled 5.3 × 10^3^ virus-associated contigs. We identified 12 novel RNA viruses from two species, *Perinereis aibuhitensis* and *P. wilsoni*, which were classified into four major viral groups: *Picobirnaviridae*, *Marnaviridae*, unclassified *Picornavirales*, and unclassified *Bunyavirales*. Our findings revealed the hidden diversity of viruses and genome structures in *Perinereis*, enriching the RNA virosphere and expanding the host range of *Picobirnaviridae, Marnaviridae*, and *Bunyavirales*. This study also highlighted the potential biosecurity risk of the novel viruses carried by *Perinereis* to aquaculture.

## 1. Introduction

Nereididae is a group of morphologically diverse marine Annelida polychaeta animals with high species diversity, including more than 45 genera and 719 species [1]. They are extensively distributed in intertidal zones, freshwater areas, and deep-sea environments, and are predominantly found in coastal areas [1]. *Perinereis*, representing the second most abundant genus within Nereididae, comprises 94 species [2,3,4,5]. *Perinereis* plays a crucial role in aquaculture hatcheries and recreational fisheries. *Perinereis* also serves as an effective model organism for absorbing and purifying environmental contaminants [6]. Previous studies have shown that the bioturbation effect of *P*. *aibuhitensis* could increase the oxygen distribution in the sediment, reduce ammonia nitrogen, sulfide, and other harmful substances in the water body, and optimize the bottom condition [6,7], playing an important role in improving the aquaculture environment. Furthermore, *Perinereis* also plays a pivotal role in the natural food chain and serves as an important component of the diet of fish and shorebirds in intertidal zones [8]. *Perinereis* is believed to be rich in nutrients, including unsaturated fatty acids, proteins, and hormones, which promote oocyte maturation and sperm production, thereby further enhancing larval shrimp production [9,10]. *P. aibuhitensis* is considered the premier source of unsaturated fatty acids for aquaculture broodstock, owing to its high content of eicosapentaenoic acid (EPA) and docosahexaenoic acid (DHA) [8,11]. Consequently, *Perinereis* is regarded as a premium live feed for broodstock in the aquaculture industry [12,13].

Studies have shown that polychaetes are the vector of shrimp white spot syndrome virus (WSSV) [14,15]. There are many examples of economic losses to the aquaculture industry caused by feeding with virus-carrying *Perinereis* [16,17,18]. Current research predominantly focuses on the detection of known viruses in economically significant aquaculture species, such as shrimp. Some studies have shown that known high-risk viruses, such as WSSV, decapod iridescent virus 1 (DIV1), and covert mortality nodavirus (CMNV), are detected in *Perinereis* [19,20,21,22,23,24]. It was also reported that viruses had been previously found in another polychaetes genus, *Nereis*. For example, Devauchelle reported the discovery of *Nereis* iridescent virus (NIV) from *Nereis diversicolor* through electron microscopy [25]. However, the exploration of unknown viruses within the *Perinereis* genus remains limited [18,19,20,21,22,23,24,25,26,27,28,29]. This gap in knowledge underscores the importance of preemptively identifying and understanding novel *Perinereis* viruses to mitigate potential economic losses due to disease outbreaks.

The application of high-throughput technology has greatly advanced our understanding of viruses. Compared with the use of traditional molecular biology technology to discover viruses, high-throughput sequencing is faster and more efficient, and plays a pivotal role in the rapid assembly and annotation of the genomes of species, as well as in the discovery of microorganisms within host samples [30,31,32]. Meta-transcriptome, a whole-transcriptome shotgun sequencing technology, can quickly generate a large number of RNA sequences and broadly identify the RNA of all viruses, including known and novel viruses [33]. The employment of high-throughput sequencing has greatly advanced our understanding of virus diversity.

In this study, we employed meta-transcriptomic sequencing to identify novel RNA viruses associated with *Perinereis*. This study will help us to better understand the hidden world of viruses in *Perinereis* and the potential impact of *Perinereis* on aquaculture.

## 2. Materials and Methods

### 2.1. Sample Collection and Processing

Specimens for this investigation were obtained from six individuals of the genus *Perinereis* without obvious clinical signs, which were collected from the same polychaetes farm in Dongfang City, Hainan Province, China, on 21 September 2018. They comprised two species within the Nereididae family, *P. aibuhitensis* (*n* = 3) and *P. wilsoni* (*n* = 3). Each intact individual sample was surface-rinsed with physiological saline at the time of collection to exclude the influence of environmental contaminants, and then they were thoroughly homogenized and total RNA was extracted using the *Trizol* reagent (Invitrogen, Carlsbad, CA, USA), according to the manufacturer’s instructions.

### 2.2. RNA Library Construction and Meta-Transcriptomic Sequencing

After passing the quality control, the extracted total RNA was used for long non-coding RNA (lncRNA) library construction and meta-transcriptomic sequencing as previously described [34,35]. The Ribo-Zero™ kit (Epicentre, Madison, Wisconsin, USA) was used to remove unnecessary rRNA from total RNA, and RNA libraries were subjected to 150 nt paired-end read sequencing and then performed on the Illumina Hiseq platform (Illumina, San Diego, California, USA). The library construction and sequencing process were performed by Novogene (Beijing, China). Each library corresponded to an individual sample, resulting in three high-throughput libraries for *P. aibuhitensis* (GW1, GW2, and GW3) and three for *P. wilsoni* (SW1, SW2, and SW3).

### 2.3. Sequence Assembly and RNA Virus Discovery

Sequence assembly and RNA virus discovery were conducted for each library, following methods outlined in our prior publications [34,35]. Briefly, we used Fastp (version 0.21.0, HaploX Biotechnology Co., LTD, Shenzhen, China, 2018) [36] to control the quality of the raw data and Trinity to assemble the reads to obtain contigs. They were then compared with the non-redundant nucleotide and protein databases downloaded from GenBank by BLASTn and BLASTx to obtain potential viral contigs. False positives were excluded by the predicted genome structure, ORF prediction, and read mapping. Furthermore, Bowtie2 (version 2.3.5.1, Center for Bioinformatics and Computational Biology, Institute for Advanced Computer Studies, University of Maryland, College Park, Maryland, USA, 2012) [37], Samtools (version 1.9, Sanger Institute, Wellcome Genome Campus, Hinxton, Cambridgeshire, CB10 1SA. UK, 2018), and Geneious Prime (version 2022.0.2, Biomatters Ltd. Auckland, New Zealand, 2022) were used to map and extend target viruses to obtain a near-complete virus sequence. The abundance of viruses in each library was expressed by calculating reads per million mapped reads (RPM) and reads per kilobase of transcript per million mapped reads (RPKM).

### 2.4. Genome Structure Prediction and Phylogenetic Analysis

This study used the ORFfinder [38] to predict potential ORFs, which start with ATG and encode proteins of more than 100 amino acids (aa). CD-search [39], Pfarm [40], and Interpro [41] were used to predict the RNA-dependent RNA polymerase (RdRp) conserved domain of the viral sequence. Through multiple sequence alignments of the aa sequences of complete RdRp coding regions, sequences with identities of less than 90% [42] were considered to be potential novel virus species.

A multiple sequence alignment of the RdRp aa sequences of the *Perinereis* RNA viruses was performed using MAFFT (version 7.490, Research Institute for Microbial Diseases, Osaka University, Suita City, Osaka Prefecture, Japan, 2013) [43] with the LINS-i algorithm. TrimAl (version 1.2, Comparative Genomics group, Bioinformatics and Genomics Programme, Centre for Genomic Regulation, Barcelona, Spain, 2009) [44] was used to trim the sequences of the multiple sequence alignment. Phylogenetic analyses and the selection of the best-fitting model were performed using IQ-tree (version 2.1.4, Center for Integrative Bioinformatics Vienna, Max F. Perutz Laboratories, University of Vienna, Medical University of Vienna, Vienna, Austria, 2015) [45], with 1000 bootstrap replicates. The best-fitting model for phylogenetic analyses was LG + I + G.

## 3. Results

### 3.1. Composition and Diversity of the Perinereis Virome

After quality control, a total of 2.6 × 10^8^ clean reads were obtained from the six high-throughput libraries, which were *de novo* assembled, and BLASTx identified 5.3 × 10^3^ suspected virus-related contigs. Through the contig length, coverage, and other parameters of these virus sequences, we obtained 24 viral conserved sequences after removing redundant sequences, including 22 complete RdRp coding regions and two viral sequences encoding the N-terminal conserved region of hantavirus glycoprotein Gc (Table S1). Through a multiple sequence alignment of the aa sequences of 22 complete RdRp coding regions, we found that the aa identities of these domains and known viral domains were less than the threshold of 90% [42], and were considered to be potential novel virus species. Finally, a total of 12 viruses were obtained, including a segmented virus. These viruses belonged to four viral families: *Picobirnaviridae* (*n* = 1), unclassified *Bunyavirales* (*n* = 1), *Marnaviridae* (*n* = 7), and unclassified *Picornavirales* (*n* = 3) (Table S1).

These twelve viruses were categorized into three groups: ten viruses belong to positive-sense single-stranded RNA viruses, one virus belongs to negative-sense single-stranded RNA viruses, and one virus belongs to double-stranded RNA viruses (Table S1). The coverage of the novel RdRps identified here ranged from 11 to 906, and the abundance of different viruses in the six libraries was calculated based on RPKM and RPM in the ranges of 0.9–98.9 and 1.9–903.4, respectively (Table S1). BLASTp analysis of the complete RdRp protein for each virus revealed that the identities between the 12 novel viruses and the best hit for each virus ranged from 26.7% to 83.9%.

**Figure 1:**
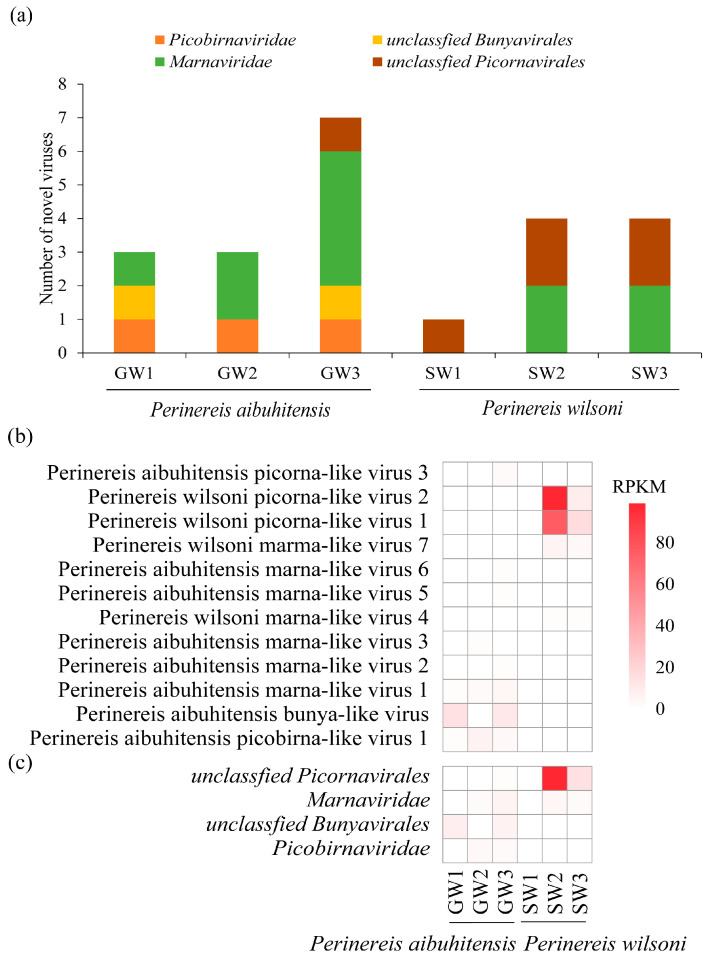
Virus abundance and distribution in the six high-throughput libraries. (**a**) The distribution of the 12 viruses identified in this study in the libraries. The *x*-axis represents the six library names, and the *y*-axis represents the number of viruses. (**b**) The abundance of the 12 viruses. (**c**) The abundance of the four major viral families in the libraries. The red scale on the right side represents RPKM.

Eight novel viruses were discovered in *P. aibuhitensis* and four in *P. wilsoni*. *P. aibuhitensis* carried viruses from four viral families: *Picobirnaviridae*, unclassified *Bunyavirales*, *Marnaviridae*, and unclassified *Picornavirales*. The viruses found in *P. wilsoni* only included two virus categories: *Marnaviridae* and unclassified *Picornavirales*. Viruses from *Marnaviridae* and *Picornavirales* were presented in both species of *Perinereis*, with marna-like viruses distributed in two species and five libraries (Figure 1a). Picorna-like viruses have higher viral abundance than viruses from other families identified from *Perinereis*. Among the 12 viruses we identified, the RPKM and RPM values of *Perinereis wilsoni* picorna-like virus 1 and *Perinereis wilsoni* picorna-like virus 2 were relatively high. *Perinereis wilsoni* picorna-like virus 1 was detected in both the SW2 and SW3 libraries, with its RPKM value reaching up to 75.8 and RPM up to 692.9. *Perinereis wilsoni* picorna-like virus 2 was detected in three libraries, SW1, SW2, and SW3, with its RPKM values reaching up to 98.9 and RPM up to 903.4 (Figure 1b, Table S1). The abundance of viruses of the *Picornavirales* lineage (*Perinereis wilsoni* picorna-like virus 1 and *Perinereis wilsoni* picorna-like virus 2) carried by *P. wilsoni* was higher than that of *P. aibuhitensis* (Figure 1b,c). However, two viruses were found exclusively in *P. aibuhitensis*: Bunya-like virus in two libraries, and Picobirna-like virus in three libraries (Figure 1a).

### 3.2. Phylogenetic Analysis of the Novel Viruses in the Order Picornavirales

**Figure 2:**
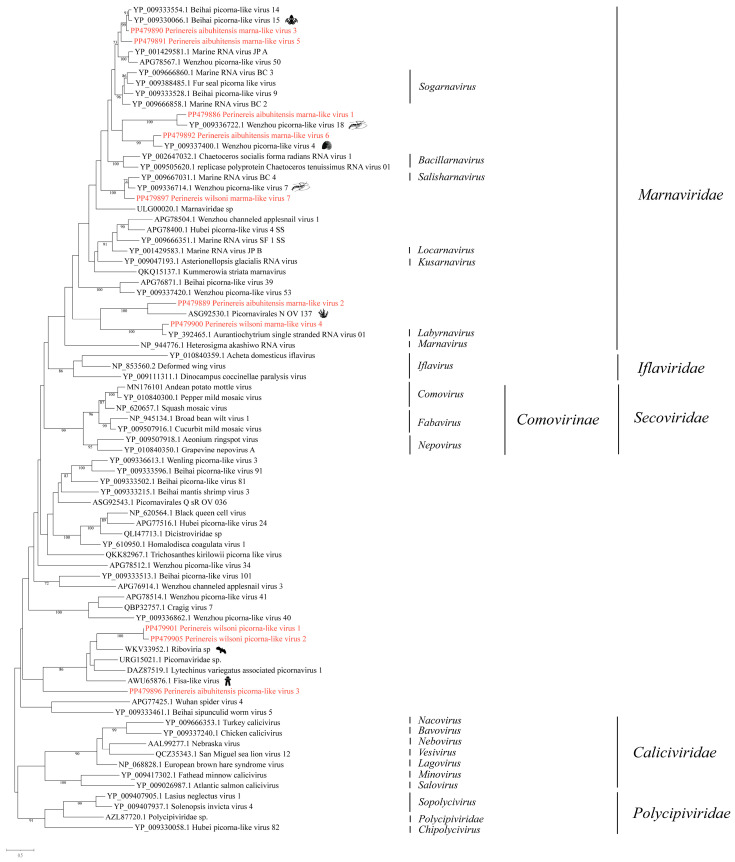
Phylogenetic analysis of representative viruses in the order *Picornavirales*. The phylogenetic tree was constructed using MAFFT for sequence alignment, trimAl for sequence trimming, and then IQ-tree to identify the LG + I + G model as the best fit for constructing the *Picornavirales* phylogenetic tree. Only bootstrap values greater than 70.0% were shown. Novel viruses obtained from *Perinereis* were marked in red. The host of the viruses most closely related to the newly identified *Perinereis* viruses was provided as a cartoon.

We obtained ten RNA viruses associated with *Picornavirales*, including seven marna-like viruses and three unclassified picornaviruses (Figure 2).

#### 3.2.1. Phylogenetic Analysis of the Novel Viruses in the Family *Marnaviridae*

From *Perinereis* samples, we identified seven RNA viruses related to the *Marnaviridae*, namely *Perinereis aibuhitensis* marna-like virus 1, *Perinereis aibuhitensis* marna-like virus 2, *Perinereis aibuhitensis* marna-like virus 3, *Perinereis wilsoni* marna-like virus 4, *Perinereis aibuhitensis* marna-like virus 5, *Perinereis aibuhitensis* marna-like virus 6, and *Perinereis wilsoni* marma-like virus 7. Five viruses were identified in *P. aibuhitensis,* and two were in *P. wilsoni*.

The best hit in the BLASTx analysis of the seven viruses revealed that RdRp aa sequence identities ranged from 44.1% to 83.9%. In order to further study the phylogenetic relationship of the seven viruses, phylogenetic analysis was performed using representative RdRp aa sequences from viruses recognized by the International Committee on Taxonomy of Viruses (ICTV) and unclassified *Marnaviridae* viruses (Figure 2). The seven novel viruses were placed in different branches within the *Marnaviridae*, thereby further revealing the diversity of the *Marnaviridae*.

In detail, *Perinereis aibuhitensis* marna-like virus 1 and Wenzhou picorna-like virus 18 (accession: YP_009336722.1) formed a distinct branch, with the RdRp protein identity of 68.8%. *Perinereis wilsoni* marma-like virus 7 and Wenzhou picorna-like virus 7 (accession: YP_009336714.1) were closely related, with the aa sequence identity in the RdRp protein reaching 70.5% (Table S1). *Perinereis wilsoni* marna-like virus 7 was tentatively determined to be within *Marnaviridae*. The aa sequence identity between *Perinereis aibuhitensis* marna-like virus 6 and Wenzhou picorna-like virus 4 (accession: YP_009337400.1) in the conserved domain was 65.2% (Figure 2, Table S1).

In addition, we revealed that the first BLASTx hit for *Perinereis aibuhitensis* marna-like virus 5 and *Perinereis aibuhitensis* marna-like virus 3 was Beihai picorna-like virus 15 (accession: YP_009330066.1), indicating that the two novel viruses were closely related. The close genetic and phylogenetic relationship further substantiated our conclusion. Phylogenetic analysis revealed that *Perinereis aibuhitensis* marna-like virus 3, Beihai picorna-like virus 15, and Beihai picorna-like virus 14 formed a distinct branch, which subsequently clustered with *Perinereis aibuhitensis* marna-like virus 5 (Figure 2). In fact, *Perinereis aibuhitensis* marna-like virus 3 and Beihai picorna-like virus 15 exhibited a high aa sequence identity of 83.9% in the RdRp conserved domain (Table S1).

*Perinereis aibuhitensis* marna-like virus 2 and *Perinereis wilsoni* marna-like virus 4 were evolutionarily distant to the other five marna-like viruses (Figure 2). These two viruses were closely associated with viruses in the unclassified *Picornavirales*. *Perinereis aibuhitensis* marna-like virus 2 and Picornavirales N_OV_137 (accession: ASG92530.1), discovered in the diatom, formed a distinct cluster branch. However, the RdRp protein aa sequence identity was only 44.1%. The aa sequence identity between the intact RdRp protein of *Perinereis wilsoni* marna-like virus 4 and Aurantiochytrium single-stranded RNA virus 01 (accession: YP_392465.1), which infects *Schizochytrium* sp., a unicellular eukaryote species in the family Thraustochytriaceae, was 81.6% (Figure 2, Table S1).

#### 3.2.2. Phylogenetic Analysis of the Novel Viruses in Unclassified *Picornavirales*

Our research identified three novel viruses within the unclassified *Picornavirales*: two from *P. wilsoni*, named *Perinereis wilsoni* picorna-like virus 1 and *Perinereis wilsoni* picorna-like virus 2, and one from *P. aibuhitensis*, named *Perinereis aibuhitensis* picorna-like virus 3.

BLASTx analysis of the intact RdRp conserved domain of *Perinereis aibuhitensis* picorna-like virus 3 showed that the best hit was the Fisa-like virus (accession: AWU65876.1), which was consistent with the results of phylogenetic analysis, though with a low aa sequence identity of 26.7% in the intact RdRp protein. BLASTx analysis of the conserved domains of *Perinereis wilsoni* picorna-like virus 1 and *Perinereis wilsoni* picorna-like virus 2 revealed that the most similar strains for both viruses were *Riboviria* sp. (accession: WKV33952.1) identified from birds. Further phylogenetic analysis revealed that *Perinereis wilsoni* picorna-like virus 1 and *Perinereis wilsoni* picorna-like virus 2 clustered together and were subsequently grouped with *Riboviria* sp. (accession: WKV33952.1). The aa sequence identities of the intact RdRp proteins between the two viruses and *Riboviria* sp. were 39.7% and 39.1%, respectively (Figure 2, Table S1).

### 3.3. Phylogenetic Analysis of the Novel Viruses in the Family Picobirnaviridae

**Figure 3:**
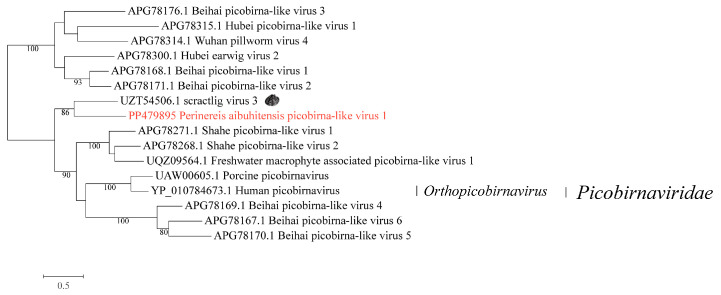
Phylogenetic analysis of the novel virus in the family *Picobirnaviridae*. The phylogenetic tree was constructed using MAFFT for sequence alignment, trimAl for sequence trimming, and then an IQ-tree to identify the LG + I + G model as the best fit for constructing the *Picobirnaviridae* phylogenetic tree. Only bootstrap values greater than 70.0% were shown. Novel viruses obtained from *Perinereis* were marked in red. The host of the virus most closely related to the newly identified *Perinereis* virus was provided as a cartoon.

One virus identified from *P. aibuhitensis* fell within the family *Picobirnaviridae*, which was named *Perinereis aibuhitensis* picobirna-like virus 1. The best hit of the BLASTx analysis of the conserved domains was scractlig virus 3 (accession: UZT54506.1) from freshwater mussels. Phylogenetic analysis showed that *Perinereis aibuhitensis* picobirna-like virus 1 and scractlig virus 3 clustered together, sharing an aa sequence identity of 35.3% in the RdRp protein (Figure 3, Table S1).

### 3.4. Phylogenetic Analysis of the Novel Viruses in Unclassified Bunyavirales

**Figure 4:**
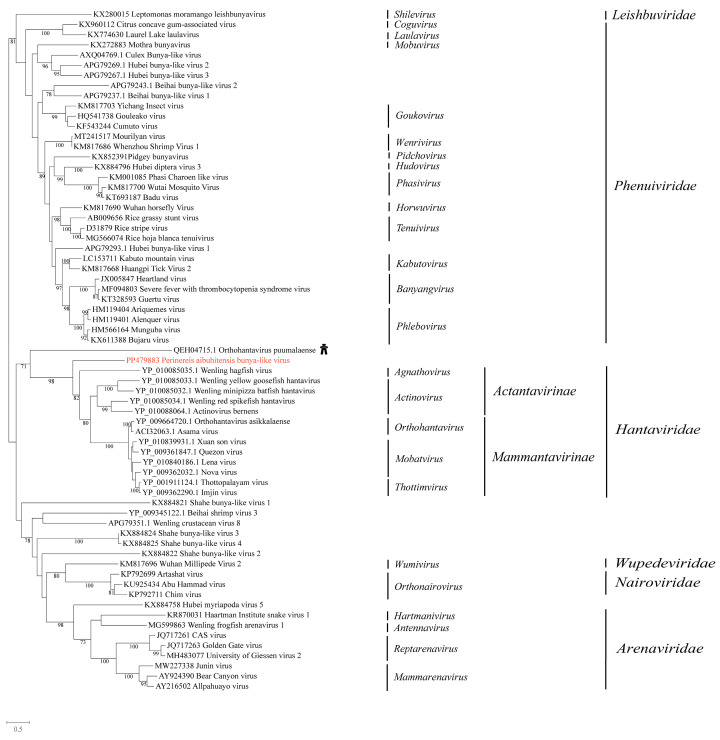
Phylogenetic analysis of the novel virus in the order *Bunyavirales*. The phylogenetic tree was constructed using MAFFT for sequence alignment, trimAl for sequence trimming, and then an IQ-tree to identify the LG + I + G model as the best fit for constructing the *Bunyavirales* phylogenetic tree. Only bootstrap values greater than 70.0% were shown. Novel viruses obtained from *Perinereis* were marked in red. The host of the virus most closely related to the newly identified *Perinereis* virus was provided as a cartoon.

We obtained a bunyavirus from *P. aibuhitensis*, which was named *Perinereis aibuhitensis* bunya-like virus. The top hit in the BLASTx search was Orthohantavirus puumalaense (accession: QEH04715.1), a virus initially discovered in humans with human immunodeficiency virus infection. A comparison of the conserved domains between *Perinereis aibuhitensis* bunya-like virus and Orthohantavirus puumalaense revealed aa identity of 27.7%. A phylogenetic analysis of the RdRp domain indicated that *Perinereis aibuhitensis* bunya-like virus clustered with viruses of the family *Hantaviridae* (Figure 4).

### 3.5. Genome Structures of the Perinereis Viruses

**Figure 5:**
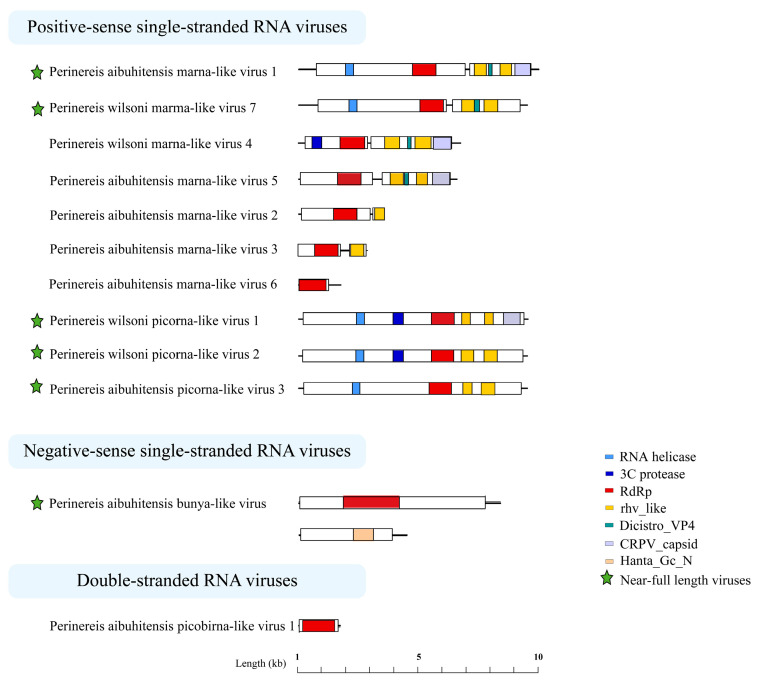
Predicted genome structures of the 12 novel viruses identified from *Perinereis* spp. Seven conserved domains were predicted from 12 novel viruses obtained from *Perinereis* spp., annotated with seven colors. Each conserved domain corresponds to a color. RdRp: RNA-dependent RNA polymerase.

The *Perinereis* virus genome structures were diverse. Most members of the order *Picornavirales* have a positive-sense, single-stranded RNA molecule between 7000 and 12,500 nt in length. Five viruses were found in this study that belong to the family *Marnaviridae*, order *Picornavirales*. Three of them, including *Perinereis aibuhitensis* marna-like virus 1, *Perinereis wilsoni* marna-like virus 4, and *Perinereis wilsoni* marma-like virus 7, had a near-complete genome sequence between 8000 and 9200 nt in length, consisting of a 5′-Untranslated Region (UTR), ORF1, ORF2, and a 3′-UTR. ORF1 encoded the RNA helicase, 3 C_protease, and non-structural proteins associated with RdRp. ORF2 encoded rhv_like, Dicistrio_VP4, CRPV_capsid, and other structural proteins, which were also possessed by viruses in the family *Marnaviridae* [46] (Figure 5).

At the same time, we identified three viruses belonging to unclassified *Picornavirales* with near-complete genome structures, ranging from 9100 nt to 9200 nt in length. These viruses exhibited genome structures similar to those of viruses of the order *Picornavirales* [47], yet with slight differences. The ORFs of *Perinereis aibuhitensis* picorna-like virus 3 encoded RNA helicase, RdRp, and two rhv_like proteins, sharing the same genome structure as the Fisa-like virus (accession: AWU65876.1) found in human feces [48]. *Perinereis wilsoni* picorna-like virus 2 and *Perinereis aibuhitensis* picorna-like virus 3 were similar in overall genome structure. The difference was that the former encoded an additional 3C_protease domain, besides an RNA helicase, an RdRp, and two rhv_like proteins (Figure 5). Of particular note, *Perinereis wilsoni* picorna-like virus 2 had the highest abundance among the 12 viruses we identified, with a RPKM value of 98.9. *Perinereis wilsoni* picorna-like virus 1 also had a high abundance, with a RPKM value of 75.8. Compared with the structure of *Perinereis aibuhitensis* picorna-like virus 3, this virus encoded two more domains, i.e., 3C_protease and CRPV_capsid. Similarly, compared with *Perinereis wilsoni* picorna-like virus 2, it encoded one additional CRPV_capsid domain. These results indicated that the picorna-like viruses obtained from *Perinereis* had distinct genome structures.

In addition, we identified two segments from *Perinereis aibuhitensis* bunya-like virus, each encoding a distinct ORF. The L segment with a length of 8066 nt encoded the RdRp conserved domain between positions 1772 and 4006 nt; similarly, the M segment with a length of 4327 nt encoded the N-terminal conserved region of hantavirus glycoprotein Gc between positions 2178 and 2929 nt (Figure 5). This was consistent with the virus structure of the family *Hantaviridae*, published by ICTV [49].

## 4. Discussion

The field of marine virology is experiencing a rapid expansion, drawing increased attention due to its critical implications for marine industry safety and the health standards of aquatic products. The introduction of high-throughput sequencing technologies has revolutionized the discovery and characterization of novel marine viruses, marking a significant advancement in our understanding of marine virology [31,33,50]. *Perinereis*, commonly used as live feed in aquaculture, has emerged as a potential vector for virus transmission [8,11,12,13]. Research has established connections between polychaetes and viruses such as the WSSV, highlighting the intricate relationships within marine ecosystems [14,15]. The presence of *Perinereis* carrying - virus, such as that of WSSV, DIV1, and CMNV, has been associated with economic losses in aquaculture, underscoring the importance of studying virus transmission dynamics in marine environments [16,17,18,19,20,21,22,23,24]. While current research predominantly focuses on known viruses affecting aquaculture species [18,19,20,21,22,23,24], there remains a significant gap in the exploration of unknown *Perinereis* viruses. This underscores the urgent need to identify and comprehend these viruses to mitigate the risk of disease outbreaks and subsequent economic losses in the aquaculture industry. Transcriptomic approaches have emerged as powerful and efficient tools for virus discovery, offering a promising avenue for studying *Perinereis* diseases. However, their application in this context, particularly in the realm of bioinformatics, has been underutilized. Further exploration and utilization of transcriptomic tools hold great potential for enhancing our understanding of marine virology and improving disease management strategies in aquaculture settings.

In this study, we leveraged meta-transcriptomic sequencing to unearth a suite of novel viruses within *Perinereis*, enriching our understanding of their virome and providing crucial data to preemptively address potential biosecurity risks. The identification of 12 novel RNA viruses within *Perinereis* spp. significantly broadens the scope of the known virome associated with these annelids. Although there is no clear pathogenic risk among the 12 novel viruses identified, the detection of these viruses in both *P. aibuhitensis* and *P. wilsoni* raises the specter of interspecies transmission and necessitates further investigation into their pathogenic potential and implications for aquaculture. In addition, combined with the previous paper [51] and private communication between the authors, we further analyzed of the correlation between the RPM value of RNA viruses and copies resulting from the fluorescence quantitative PCR Ct value, and regressed a trend relationship between RPM and virus copies (VCs), which is VCs (copies/ng-RNA) = 0.7401 × RPM^0.8621^. Considering that higher viral loads in the host are more likely caused by infection, we speculated that a virus with an RPM value greater than 300 may be equivalent to a virus load above 100 copies/ng-RNA in the tissue, of which the presence of the virus may be considered an infection but not a contamination. In our present study, the RPM values of two viruses, i.e., *Perinereis wilsoni* picorna-like virus 1 and *Perinereis wilsoni* picorna-like virus 2, were above 600, which suggested that these two viruses might be highly copied in the samples. However, more experiments are needed to confirm the activity of these viruses and to further assess the risk.

The wide geographical distribution of *Perinereis* spp. spans the Indo-Pacific to the southeastern Atlantic [52,53]. The presence of diverse RNA viruses in *P. aibuhitensis*, a dominant species in intertidal zones, hints at the potential for widespread viral dissemination through the food chain, facilitated by ocean currents. Moreover, the increasing reliance on aquaculture and the trade of aquatic products, including the use of *Perinereis* as a live feed, may inadvertently amplify the risk of viruses spread across international borders [16,54]. The limited current understanding of *Perinereis* viruses serves as a reminder of the vast, uncharted virome that awaits discovery and characterization. This study not only contributes to the foundational knowledge of *Perinereis* virology, but also emphasizes the need for a proactive approach to managing the health of our marine ecosystems and the sustainability of our aquaculture industries.

## 5. Conclusions

In this study, we identified and characterized 12 novel RNA viruses from *Perinereis*, spanning four major viral families: *Picobirnaviridae*, *Marnaviridae*, and unclassified members of *Picornavirales* and *Bunyavirales*. Our research uncovered the tip of the iceberg of the RNA virome diversity within *Perinereis*. The discovery of these novel viruses provides critical insights into the biosecurity risks of using *Perinereis* in aquaculture.

## Data Availability

The raw data obtained from the meta-transcriptomic sequencing conducted in this study can be accessed at the NCBI Sequence Read Archive (SRA) database with the BioProject accession number PRJNA1088991. The viral sequences derived from this study have been deposited in GenBank with the accession numbers PP479882-PP479905 (Table S1).

## Supplementary Materials

The following supporting information can be downloaded at https://www.mdpi.com/article/10.3390/vetsci11060273/s1, Table S1: Diversity of viruses in *Perinereis*.
